# Exploring a route to a selective and sensitive portable system for explosive detection– swab spray ionisation coupled to of high-field assisted waveform ion mobility spectrometry (FAIMS)

**DOI:** 10.1016/j.fsisyn.2019.07.009

**Published:** 2019-08-27

**Authors:** C. Costa, E.M. van Es, P. Sears, J. Bunch, Vladimir Palitsin, H. Cooper, M.J. Bailey

**Affiliations:** a Ion Beam Centre, University of Surrey, Guildford, Surrey, GU2 7XH, UK; b National Physical Laboratory, Teddington, Middlesex, TW11 0LW, UK; c Defence Science and Technology Laboratory, Sevenoaks, Kent, TN14 7BP, UK; d University of Birmingham, Edgbaston, Birmingham, B15 2TT, United Kingdom; e Department of Chemistry, University of Surrey, Guildford, Surrey, GU2 7XH, UK

**Keywords:** Explosives, Swab spray, Mass spectrometry, FAIMS

## Abstract

Paper spray mass spectrometry is a rapid and sensitive tool for explosives detection but has so far only been demonstrated using high resolution mass spectrometry, which bears too high a cost for many practical applications. Here we explore the potential for paper spray to be implemented in field applications with portable mass spectrometry. This involved (a) replacing the paper substrate with a swabbing material (which we call “swab spray”) for compatibility with standard collection materials; (b) collection of explosives from surfaces; (c) an exploration of interferences within a ± 0.5 *m/*z window; and (d) demonstration of the use of high-field assisted waveform ion mobility spectrometer (FAIMS) for enhanced selectivity. We show that paper and Nomex® are viable collection materials, with Nomex providing cleaner spectra and therefore greater potential for integration with portable mass spectrometers. We show that sensitive detection using swab spray will require a mass spectrometer with a mass resolving power of 4000 or more. We show that by coupling the swab spray ionisation source with FAIMS, it is possible to reduce background interferences, thereby facilitating the use of a low resolving power (e.g. quadrupole) mass spectrometer.

## 1 Introduction

Screening techniques capable of rapidly detecting explosive compounds play an essential safeguarding role in areas recognised as being at “high-risk” of terrorist activities. Current methods that are widely implemented for screening explosives are based on thermal desorption coupled to ion mobility spectrometry (TD-IMS) [[1], [2], [3], [4]]. The thermal desorption process, however, can offer unsatisfactory performance for thermally labile compounds of interest which break down upon heating [[4], [5], [6], [7]]. We have previously shown how paper spray, a rapid ionisation technique previously used in the analysis of biofluids [[8], [9], [10], [11], [12], [13], [14], [15], [16], [17], [18], [19]], ink [20] and foodstuffs [[21], [22], [23], [24], [25]] can be used as an effective and efficient alternative to TD-IMS for the analysis of explosive compounds at ultra-trace levels (25 pg) [26]. Paper spray can detect multiple explosive compounds including trinitrotoluene (TNT), 1,3,5-trinitroperhydro-1,3,5-triazine (RDX), octahydro-1,3,5,7-tetranitro-1,3,5,7-tetrazocine (HMX), pentaerythritol tetranitrate (PETN), tetryl, nitroglycerin (NG), tetryl, picric acid (PA) and hexamethylene triperoxide diamine (HMTD) [26].

During paper spray, samples are deposited directly on to a triangular-shaped paper substrate. A voltage and a drop of solvent are applied to the back end of the paper, which extracts and sweeps analytes from the substrate and induces a spray which is directed into a mass spectrometer for detection. It has been demonstrated by other groups that the substrate from which the spray is induced does not necessarily need to be paper. Alteration of the substrate has previously provided many other techniques, which are similar to paper spray such as leaf spray [27,28] or tissue spray (from a needle tip) [29], which are far more suited to their desired application.

Current techniques for the screening of explosives generally involve swabbing of the surface with a collection material such as cotton, Nomex® or Teflon coated fibreglass. These materials are employed in explosives screening because they are known to be efficient at picking up relevant materials from surfaces [30]. Bain et al. [31] have recently shown that swab touch spray (using a cotton swab) can be used to pick up explosives from surfaces such as gloves and human skin. Swab touch spray utilises a different substrate, geometry and solvent delivery system to what is described here. Rather than introduce a new swabbing material as per Bain et al. [31], we explore the potential to integrate materials that are currently used in explosives screening for this application, with the aim of easing integration into the operational workflow. In our previous work [26] only a paper substrate was considered. Here we consider the use of other collection materials (Nomex, Teflon coated fibre glass and cotton) that are currently employed in security screening programmes.

Research to date on paper spray for explosives detection [26,32,33], has only considered laboratory-based mass spectrometers. However, many operational scenarios (e.g. airports, military checkpoints) cannot afford the associated high acquisition cost or footprint of such instruments. Miniature mass spectrometers are now available at a fraction of the cost of laboratory based instruments, but with a lower mass resolution [34]. Therefore, in this paper we use a high-resolution mass spectrometer to explore interferences within a ±0.5 *m/z* range of analyte peaks to facilitate integration with portable mass spectrometry. We also explore the use of high-field assisted waveform ion mobility spectrometer (FAIMS) [35] to improve the selectivity of the analytical method.

## 2 Experimental

A paper spray source was designed and built in-house as described previously [26,44]. This source was coupled to a Thermo Scientific™ Q Exactive™ Hybrid Quadrupole-Orbitrap™ mass spectrometer (Thermo Scientific, Bremen, Germany). Data was acquired in full scan mode (*m/z* 100–500) with a resolution of 280,000 at *m/z* 200 and analysed using Xcalibur 2.10 software (Thermo Fisher Scientific, Bremen, Germany).

Paper spray measurements used Whatman Grade I chromatography paper as a substrate. Nomex® (meta-aramid swabs, 200 ct), Teflon coated fibreglass (PTFE coated trap, 100 ct) and cotton gloves were obtained from DSA Detection (St Albans, UK) and investigated as alternative substrates. All substrates were cut into triangles (1.6 × 2.1 cm, b × h). Aluminium foil was folded around the base of the substrate to prevent contamination of the clip supplying the voltage. The substrate was placed on a pre-cut glass slide to prevent contamination of the sample holder.

Swabbing experiments used Solmedia glass slides (Shrewsbury, UK), a generic Dell keyboard (Berkshire, UK) used in an explosive-free environment and a new “Classicline” keyboard (Trust, Netherlands) as deposition surfaces.

Explosive standards were prepared from certified reference materials of trinitrotoluene (TNT), 1,3,5-trinitroperhydro-1,3,5-triazine (RDX), octahydro-1,3,5,7-tetranitro-1,3,5,7-tetrazocine (HMX), pentaerythritol tetranitrate (PETN), tetryl, nitroglycerin (NG), tetryl, picric acid (PA) and hexamethylene triperoxide diamine (HMTD), which were obtained from AccuStandard through Kinesis (St Neots, UK). Chloramphenicol (CAM) was obtained from Sigma Aldrich (Poole, UK). Optima™ LC-MS grade solvents, methanol (MeOH) and acetonitrile (ACN), were used to prepare all solutions and solvent mixtures (Fisher Scientific, Loughborough, UK). Sodium chloride (NaCl; Sigma Aldrich, Poole, UK) and ammonium nitrate (NH_4_NO_3_; Fisher Scientific, Loughborough, UK) were used as additives to the spray solvent.

Adducts determined and in previous work [26] were used for detection of relevant analytes. The analysis method involved the addition of the analytes to the paper, followed by the addition of 5 μL of 500 ng/mL (2.5 ng) solution of CAM (prepared in MeOH), spray solvent (50 μL; 0.1 mM NH_4_NO_3_/NaCl in 100% MeOH) and the application of a 2.0 kV spray voltage. As per our previous publication, CAM (at 2500 pg) was used as a spray monitoring tool to prevent false negative events. The internal standard threshold was set at 1 × 10^5^ counts (based on the sum intensity of CAM peaks). Any replicate measurement below this threshold was considered a failed spray [26].

The MS was operated at a capillary temperature of 90 °C and S-lens RF level of 80 in negative mode for the detection of TNT, RDX, HMX, PETN, NG, tetryl and PA. Operational parameters for HMTD were identical except for the spray voltage, which was increased to 3.5 kV.

To explore the possibility of reducing interferences in a ±0.5 *m/z* range, a FAIMS system (Owlstone, Cambridge, UK) was coupled to the Q-Exactive™ Plus Orbitrap mass spectrometer. Samples containing TNT, RDX, HMX, PETN, Tetryl, NG and PA (500 ng/mL in 0.1 mM NH_4_NO_3_/NaCl 100% MeOH) were introduced using ESI infusion (flow rate, 5 μL/min) and the dispersion and compensation voltages (DV and CV) of the FAIMS were swept across their range to produce a number of 2D scans. The parameters for the UltraFAIMS were set using a software interface provided by Owlstone (UltraFAIMS Control Software V2.00.0.00-r0) with the hardware settings fixed at an analytical gap width of 100 μm, trench length of 96 mm and chip thickness 700 μm. The chip region temperature was set to 100 °C and the bias voltage was set to 0 V.

A 2D scan was carried out over the dispersion field (DF) range of 200–300 Td and a compensation field (CF) range of -10-10 Td with a CF sweep duration of 30 s. The sensitivity for each explosive compound peaked between a DF of 210–220 Td and clear separation was observed at DF values > 270 Td. 1D sweeps were then carried out at a fixed DF (200–300 Td) and CF of −2 to 2 Td with a CF sweep time of 300 s, allowing for optimum CF values to be clearly identified.

## 3 Results and discussion

### 3.1 Substrate compatibility

Substrates made from Whatman grade 1 chromatography paper, cotton, Nomex® and teflon-coated fibreglass were spiked with 2, 10, 20, 50, 100 and 200 pg of analyte and tested for suitability. Various volumes (20–100 μL) of spray solvent (0.1 mM NH_4_NO_3_/NaCl in MeOH) and clip voltages (1–5 kV) were investigated for compatibility. None of the analytes could be detected using either the cotton or teflon-coated fibreglass substrates. However, analytes were readily detected using Nomex® and thus Nomex® was a viable alternative to paper. From this point forward, paper spray refers to the use of Whatman Grade I chromatography paper and “swab spray” refers to the use of Nomex®.

### 3.2 Detection of HMTD

**Fig. 1:**
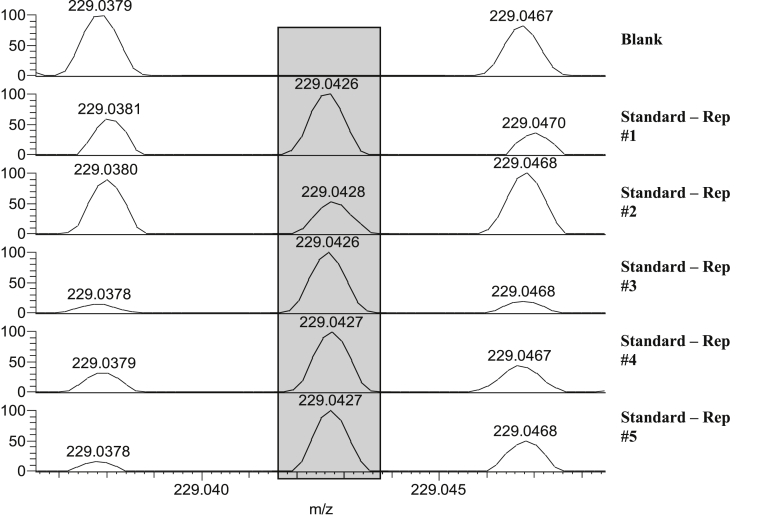
Example spectra showing the HMTD signal [HMTD-2H + Na]^+^ in blank measurements (top spectrum) and 5 replicate standard measurements using SS-MS. 2500 pg of HMTD were deposited on the substrate for analysis (5 μL of 500 ng/mL).

To show applicability of a peroxide explosive for this type of analysis, the swab spray method was modified for the detection of HMTD, which produces positive ions. The same experimental conditions were used as for the detection of the remaining seven explosives in negative ion mode, with the exception of the applied voltage, which was raised to 3.5 kV. HMTD was detected at *m/z* 229.0431 ([HMTD-2H + Na]^+^), as shown in Fig. 1.

### 3.3 Comparison of swab spray and paper spray

Solutions containing the analytes were prepared over a range of concentrations (5–180 ng/mL). The standard solutions were drop deposited (5 μL) onto the substrate and allowed to dry (c.a. 1 min)before analysis using the protocol described above.

Due to the high variability observed with paper spray [26,36], estimation of limits of detection in the absence of an isotopically labelled internal standard is not trivial. As such, for this publication we report on the lowest mass detected. This is defined as follows: in addition to the internal standard cut-off discussed above, successful detection of the analytes required a minimum of 500 counts (peak height) on at least 3 replicate measurements.

Table 1 reports the lowest detected mass for both substrates investigated (this is based on the most abundant ion for each analyte) [26]. From Table 1 it is clear that replacing the paper substrate for a swab does not result in a loss of sensitivity, with the exception of NG.

**Table 1:** Lowest mass detected in all replicate measurements for each explosive material for swab spray and paper spray for a 30 s acquisition.

	Lowest detected mass (pg)	
	Paper Spray	Swab Spray
TNT	25	25
RDX	25	25
HMX	25	25
PETN	25	25
Tetryl	25	25
NG	25	50
PA	25	25

### 3.4 Surface swabbing

An investigation was carried out to demonstrate the feasibility of collecting explosives from a surface and analysing with both paper spray and swab spray. Known masses of explosives were drop deposited (10 and 25 ng)onto glass slides). The solutions were left to dry until no residue could be observed. The surface was then swabbed using Whatman grade 1 paper or Nomex, the internal standard was added (and allowed to dry) and the substrate was mounted in the source holder for analysis. The data was considered in the same way as described above. Swab spray performed slightly better than paper spray; this is presumably due to the superiority of the swab in picking up explosive compounds from the glass surface. Thus, any further swabbing experiment was carried out using Nomex as a substrate. In Table 2, the results are also compared to Tsai et al. [33], which also used paper to collect explosives from glass slides. The method developed here demonstrates detection of explosives at two orders of magnitude than those obtained in previous work and for a wider range of explosives [33]. Bain et al. [31] recovered explosives from gloves and hands, so no direct comparison can be made, however, we have target other analytes such as tetryl, NG and PA.

**Table 2:** The lowest mass of explosives detected in each replicate measurement from various surfaces. Key: N/D = not detected.

Lowest detected mass (ng)					
	Tsai et al. [33], recovery from glass slide	This work, recovery from glass slide		Clean keyboard	Used keyboard
	Paper spray	Paper Spray	Swab Spray	Swab Spray	Swab Spray
TNT	800	N/D	N/D	ND	Partial at 25
RDX	100	25	10	10	10
HMX	600	25	10	Partial at 10	10
PETN	100	25	10	Partial at 10	10
Tetryl	–	25	10	Partial at 10	10
NG	–	N/D	N/D	ND	25
PA	–	10	10	10	10

The same methodology was used to analyse explosives drop deposited onto individual keys of a clean keyboard at two different masses (10 and 25 ng) using swab spray. Both TNT and NG were not detected at the two masses tested here and both RDX and PA were detected at 1 ng/Conversely, HMX, PETN and tetryl were only detected in 2 out of 3 replicate swabbing experiments and were therefore defined as partially detected.

**Fig. 2:**
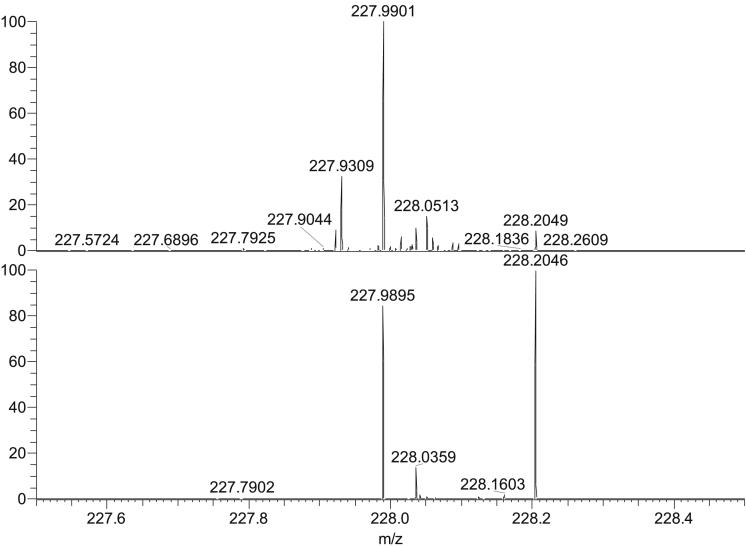
Mass spectra showing 500 pg of PA (*m/z* 227.9894) after swabbing a dirty keyboard key (top) and a clean keyboard key (bottom).

The same experiment was also carried out using a used keyboard and the data is shown in Table 2. With the exception of TNT (partially detected at 25 ng) and NG (detected at 25 ng), all analytes were successfully detected at 10 ng of material. The more successful detection can be rationalised by the higher recovery of analytes caused by the presence of dirt on the keyboard changing the surface adhesion. It was also observed that the background signals of the samples collected from the dirty keyboard were higher than those collected from the clean keyboard, as shown in Fig. 2.

### 3.5 Mass interferences

In order to produce a field-deployable technique, the mass spectrometer that the swab spray ionisation source is coupled to must be of a compact size and low cost compared with laboratory instruments. The portable MS instruments currently available are not capable of reaching the high mass resolving power of 280,000 (at *m/z* 200) used here, with the best available portable mass spectrometers only providing resolving powers in the order of 6000 [[37], [38], [39], [40]].

As a next step towards a portable system for explosives detection, we have explored the mass resolving power that is required to resolve background interferences from analyte signal for both swab spray and paper spray. To this, we have estimated the mass resolving power that would be required in order for 200 pg of analyte to be distinguished from the background at a 3:1 ratio (see Table 3).

**Table 3:** Estimated resolution required to separate the analyte signal from background peaks (>3:1) for samples containing 200 pg of each explosive compound.

Explosive	Paper Spray	Swab Spray
TNT	3400	3400
RDX	1200	1700
HMX	17,500	2800
PETN	8300	3350
Tetryl	6600	1300
NG	1750	2500
PA	4900	1350

**Fig. 3:**
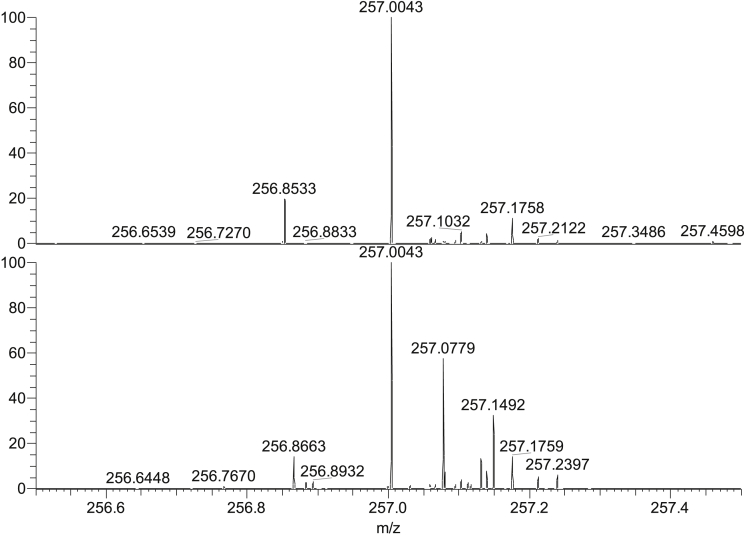
Mass spectra for RDX (200 pg, *m*/*z* ± 0.05 *m*/*z*) sprayed using swab spray (top) and paper spray (bottom).

**Fig. 4:**
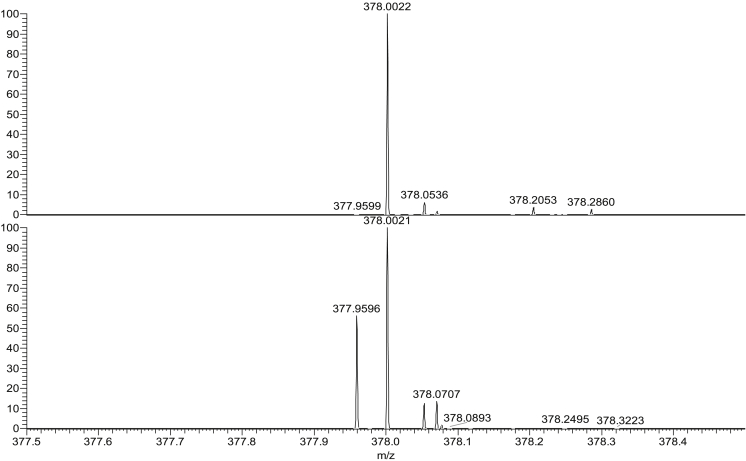
Mass spectra for PETN (200 pg, *m*/*z* ± 0.05 *m*/*z*) sprayed using swab spray (top) and paper spray (bottom).

Generally, swab spray gave cleaner background than paper spray (see Fig. 3 and Fig. 4). For swab spray, it was found that a mass resolving power of greater than 3350 was required to separate all analyte peaks from their background, compared with 17,500 for paper spray. Ion trap or ToF systems [[37], [38], [39],^41^,^42^] do therefore appear to have sufficient mass resolution to enable detection of low levels of the explosives considered here on clean Nomex swabs. It might be expected that swabbing from a dirty surface would attract more background interferences and thereby increase the mass resolution required to distinguish analytes from their background. However, Fig. 1 shows that although for a dirty keyboard the background is higher than for a clean keyboard, the closest interference to picric acid is 0.06 *m/z* away from the [M − H]^-^ peak, and so in this case a mass spectrometer with a resolution of 3800 should be able to resolve the analyte from the background. Of course, the mass resolution that would be required to discriminate all explosives without any false alarms on any dirty swab can only be determined through pseudo operational trials, because there is no “standard” dirty swab. This should be the subject of further work.

Whilst mass spectrometers with mass resolutions of 4000 or more do exist, the more affordable and field deployable types employ quadrupoles with only unit mass resolution [41,43]. Therefore we investigate whether pre-filtering of ions using FAIMS can be carried out to allow possible integration with a lower resolution system.

### 3.6 Integration of FAIMS

**Fig. 5:**
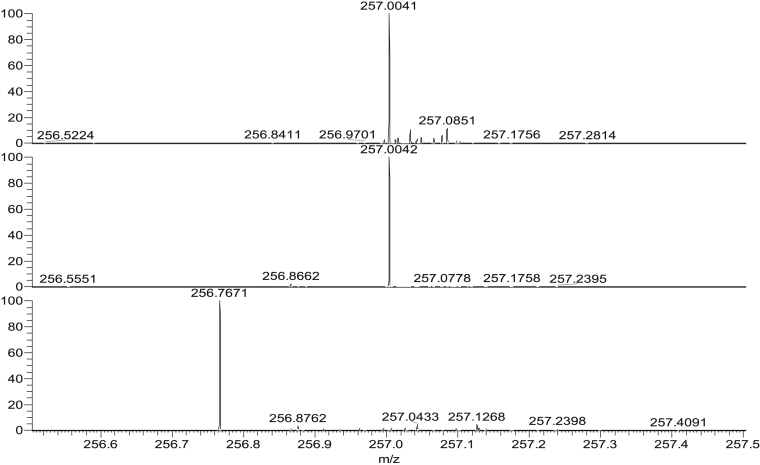
Top, swab spray-FAIMS-MS (DF 280 Td, CF 0.5 Td); Middle, swab spray-FAIMS-MS (DF 220 Td, CF 0.6); Bottom swab spray-MS of [RDX+^35^Cl]^-^ (500 pg, *m*/*z* 257.0037 ± 0.5).

Samples containing 500 pg of explosives were run using swab spray at a DF of 220 and 280 Td and at a fixed CF of 0.6 Td. These results were compared to swab spray results which were collected with no FAIMS attachment. An example is presented in Fig. 5 below, and shows complete elimination of the background signals around the signal for RDX at *m/z* 257.0043 (second panel).

The data presented here clearly shows that with the right FAIMS settings, the background can be virtually eliminated from swab spray spectra. Therefore integration of FAIMS-MS offers considerable promise for further exploitation, to enable low resolution mass spectrometry from a swab spray source.

## 4 Conclusions

Swab spray coupled to a high-resolution mass spectrometer was successfully used to detect explosive compounds including TNT, RDX, HMX, PETN, tetryl, NG and PA with the lowest detected mass below 50 pg. The lowest detected mass of HMTD was 2.5 ng. The recovery and detection of trace quantities of explosives from glass slides showed enhanced sensitivity compared with previously published work. This was extended to other surfaces, including clean and dirty keyboards, during which >25 ng of explosives could be observed, an operationally relevant sensitivity. Interferences in a ±0.5 *m/z* range were also explored in order to specify the resolution required of a field deployable mass spectrometer; this was determined to be < 4000. It was also shown that coupling with FAIMS to the swab spray source, interferences with a ±0.5 *m/z* range for the analytes of interest can be eliminated. This opens up the opportunity of using a lower resolution and thus more affordable portable quadrupole mass spectrometer for this application.

## Conflicts of interest

The authors do not have any conflicts of interest to declare.
